# Effect of the combination of remifentanil and neuromuscular blockers in pediatric endotracheal intubation: a prospective, double-blinded, randomized clinical trial

**DOI:** 10.1186/s12887-025-06010-y

**Published:** 2025-08-18

**Authors:** Lanxin Qiao, Di Zhang, Zhengzheng Gao, Tingting Zi, Jianmin Zhang, Lijing Li, Fang Wang

**Affiliations:** 1 Department of Anesthesiology, Beijing Children’s Hospital, Capital Medical University, National Center for Children’s Health, Beijing, 100045 China; 2 Department of Anesthesiology, The First Hospital of Tsinghua University, Beijing, 100016 China

**Keywords:** Pediatric, Remifentanil, Intubation-related adverse events

## Abstract

**Background:**

Endotracheal intubation must be performed more carefully in children than in adults, as children are likely to more serious consequences due to the buck, move, and hemodynamic changes during the procedure. The aim of this study was to explore whether increasing the use of remifentanil during anesthesia induction can further improve the effectiveness and safety of tracheal intubation in children.

**Design:**

This double-blind clinical trial included patients who underwent elective surgery under general anesthesia for intubation at Beijing Children’s Hospital.

**Methods:**

One hundred thirty-eight pediatric patients aged 1–12 years, classified as having American Society of Anesthesiologists (ASA) physical status I or II, who received general anesthetics for elective surgery were randomly divided into a remifentanil group (group R) (*n* = 69) and a placebo group (group C) (*n* = 69). In group R, 1 µg/kg remifentanil was intravenously infused after the intravenous infusion of other induction drugs. In group C, the same volume of normal saline was injected intravenously. The primary outcome measure was successful intubation at the first attempt with no adverse events.

**Results:**

In total, 129 patients (64 in the group R and 65 in the group C) were enrolled. The incidence of first-attempt successful tracheal intubation with no adverse events was greater in group R (87.5%, *n* = 56) than in group C (60.0%, *n* = 39) (OR, 4.7; 95% CI = 1.9–11.4; *p* < 0.001).

**Conclusions:**

Compared with the use of cisatracurium alone, the combination of low-dose remifentanil with cisatracurium was associated with a higher rate of successful first-attempt intubation without adverse events.

**Trial registration:**

Chinese Clinical Trial Registry. Retrospectively registered. Identifier: ChiCTR2400089691. Date: 13/9/2024.

## Introduction

A sufficient depth of anesthesia and degree of muscle relaxation are necessary for complication-free, successful tracheal intubation. Compared with adults, children experience more airway-related adverse events under general anesthesia [1, 2], so the anesthesia requirements for tracheal intubation are greater. To relieve tension and anxiety and prevent discomfort in children during anesthesia induction, sedatives are usually given first, and then muscle relaxants are administered. Currently, propofol and cisatracurium are commonly administered intravenously for anesthesia induction in children. Previous data revealed that the onset time of a single injection of propofol was 30–60 s, the effect peaked at approximately 120 s, and the maintenance time was approximately 10 min [3, 4]. However, according to the results of our previous study [5], the bispectral index (BIS) in most children was greater than 60 3 min after propofol injection, and the BIS in some children was greater than 60 after 90 s, indicating that the depth of anesthesia was not suitable for tracheal intubation. With cisatracurium, the depth of anesthesia was better at 120 s after injection. Therefore, in some cases, the sedative effect of propofol may not be the best when cisatracurium is used.

Remifentanil is a short-acting opioid with analgesic and partial sedative effects and is commonly administered via intravenous injection. Remifentanil is known for its rapid onset and rapid metabolism [6] and can reduce the likelihood of hemodynamic fluctuations during intubation when combined with other anesthetic drugs[7, 8]. Several studies have revealed that remifentanil improves the patient’s condition for intubation [9], as it induces anesthesia in less than 90 seconds[7–10], and can be safely used in children [11]. However, in most of these studies, muscle relaxants were not used, the dose of remifentanil was relatively high, and there were some accompanying adverse reactions. Therefore, the aim of this study was to explore whether the combination of low-dose remifentanil with common anesthetics can improve patients’ likelihood of intubation without causing adverse reactions.

## Methods

### Patients

This prospective, double-blind clinical trial was approved by the Zhang Yi of the Ethics Committee of Beijing Children’s Hospital, Capital Medical University (approval number: [2024]-Y-048-D, date of approval: 15/3/2024) and registered in the Chinese Clinical Trial Registry (retrospectively registered; registration number: ChiCTR2400089691; registration date: 13/9/2024). All the legal guardians of the children enrolled in the study signed informed consent forms before entering the operating room.

Pediatric patients aged 1–12 years who were classified as having American Society of Anesthesiologists (ASA) physical status I or II and who received general anesthetics for elective surgery at Beijing Children’s Hospital, Capital Medical University, from September 2024–December 2024 were enrolled in this study. All patients were required to fast for more than 6 h, abstain from formula milk for more than 4 h and abstain from all drinks drink for more than 2 h before surgery. Patients were excluded if they had (1) severe gastroesophageal reflux, tracheoesophageal fistula or hiatal hernia; (2) expected difficulty in tracheal intubation; (3) severe cardiovascular or respiratory diseases; (4) dysfunction of the liver or kidney; or (5) contraindications to the use of remifentanil.

### Randomization

Using SAS 9.4 statistical software, a random number table was generated to randomly allocate the participants into 2 groups: the remifentanil group (group R) and the placebo group (group C). In group R, 10 µg/ml remifentanil was prepared and administered intravenously in increments of 1 µg/kg (0.1 ml/kg) after the intravenous administration of anesthetics commonly used for induction. In group C, 0.1 ml/kg normal saline was administered. The remifentanil solution prepared was visually indistinguishable from the normal saline solution. An independent researcher prepared the solutions and randomly divided the patients into the two groups, and the other researchers and patients were blinded to the treatment groups.

### Trial intervention and anesthesia protocol

No medication was used before anesthesia. Peripheral intravenous access was established for all patients in the hospital ward. Electrocardiography (ECG), noninvasive blood pressure (NIBP), pulse oxygen saturation (SpO_2_) and the BIS were routinely monitored after the patient entered the operating room.

All patients were preoxygenated for 3 min before the induction of general anesthesia. A sedative (propofol 2–3 mg/kg, injection rate 20 ml/min), neuromuscular blocker agent (cisatracurium 0.1 mg/kg, within 5–10 s), and long-acting opioid (sufentanil 0.3 µg/kg, within 2 min) were administered sequentially. In this study, 0.1 ml/kg of the study drug was administered via intravenous injection immediately thereafter induction (for group R, 1 µg/kg remifentanil was given; for group C, the corresponding volume of normal saline was given). No other medication was used during anesthesia induction.

Endotracheal intubation was performed by an anesthesiologist with more than 2 years of experience, using the most commonly used intubation tools. If the airway was found to be difficult to manage, the ‘2022 American Society of Anesthesiologists Practice Guidelines for Management of the Difficult Airway’ was consulted. The appropriate-sized (4 + age/4, adjusted according to physical development) tracheal tube with a cuff was selected. Endotracheal intubation was initiated after the anesthesiologist determined that the patients met the requirements for intubation, that is, loss of consciousness, jaw relaxation, ease of laryngoscopy and no movement of the vocal cords. Caution was taken to avoid touching the carina during the intubation process. If intubation failed, that is, if the patient coughed or moved, which was considered to be caused by insufficient depth of anesthesia, 1–1.5 mg/kg propofol was administered to increase the depth of anesthesia, and then, intubation was attempted again.

If bradycardia affected hemodynamics during induction, the procedure was stopped immediately. If necessary, corresponding measures (intravenous atropine, adrenaline, etc.) were taken. If the condition could not be relieved, the relevant parties were unblinded to ensure patient safety. The method for maintaining anesthesia intraoperatively was determined by the responsible anesthesiologist.

### Outcomes

The primary outcome measure was successful intubation at the first attempt with no adverse events. First-attempt intubation success was defined as successful placement of the endotracheal tube in the glottis at the first attempt. The adverse events included choking, body movement and arrhythmia requiring intervention.

The secondary outcome measures included the time from induction to successful endotracheal intubation (from the start of drug administration to successful placement of the endotracheal tube in the glottis), desaturation occurring (SpO_2_ < 95%) during induction to successful intubation and the incidence of laryngospasm or bronchospasm. Hemodynamic parameters, including the mean arterial pressure (MAP) and heart rate (HR), were recorded immediately before anesthesia induction (baseline), immediately before intubation, immediately after intubation, and 5 min after intubation.

### Sample size calculation

According to the observation and preliminary statistics on the success rate of this event in daily work, and referring to some related studies. The success rate of first intubation for different devices mentioned in the study by Lacquiere D et al. [12]. were 98%, 92%, and 88%, respectively, with success rates here not involving complications and only defined as successful tracheal intubation. And the success rate of Grillot N et al. ‘s observation [17] index of “first attempt without major complications” which was similar to the purpose of this study (66.1%, 71.6%).

Therefore, sample size calculations for this trial assumed an event rate of 70% in group C and 90% in group R and 10% missing data for the primary end point. A sample size of 138 patients at a randomization ratio of 1:1 (approximately 69 randomized to group R and approximately 69 to group C) would be sufficient to show an effect with a 5% significance level and a power of 80%.

### Statistical analysis

All analyses were conducted using IBM SPSS (version 24.0, USA). Normally distributed numerical data are expressed as the means ± standard deviations (x ± s); comparisons between groups were performed via independent samples t tests. Nonnormally distributed numerical data are presented as medians and interquartile ranges, and group comparisons were performed via the Mann‒Whitney U test. Qualitative variables are presented as the number of patients (percentages), and the chi-square test or Fisher’s exact test was used. Repeated-measures ANOVA was used to analyze the MAP or HR values at multiple time points in the groups. Post hoc Bonferroni correction was used to compare differences at the same time points in different groups and at different time points in the same groups. A *p* value < 0.05 indicated statistical significance.

## Results

### Trial population

**Fig. 1:**
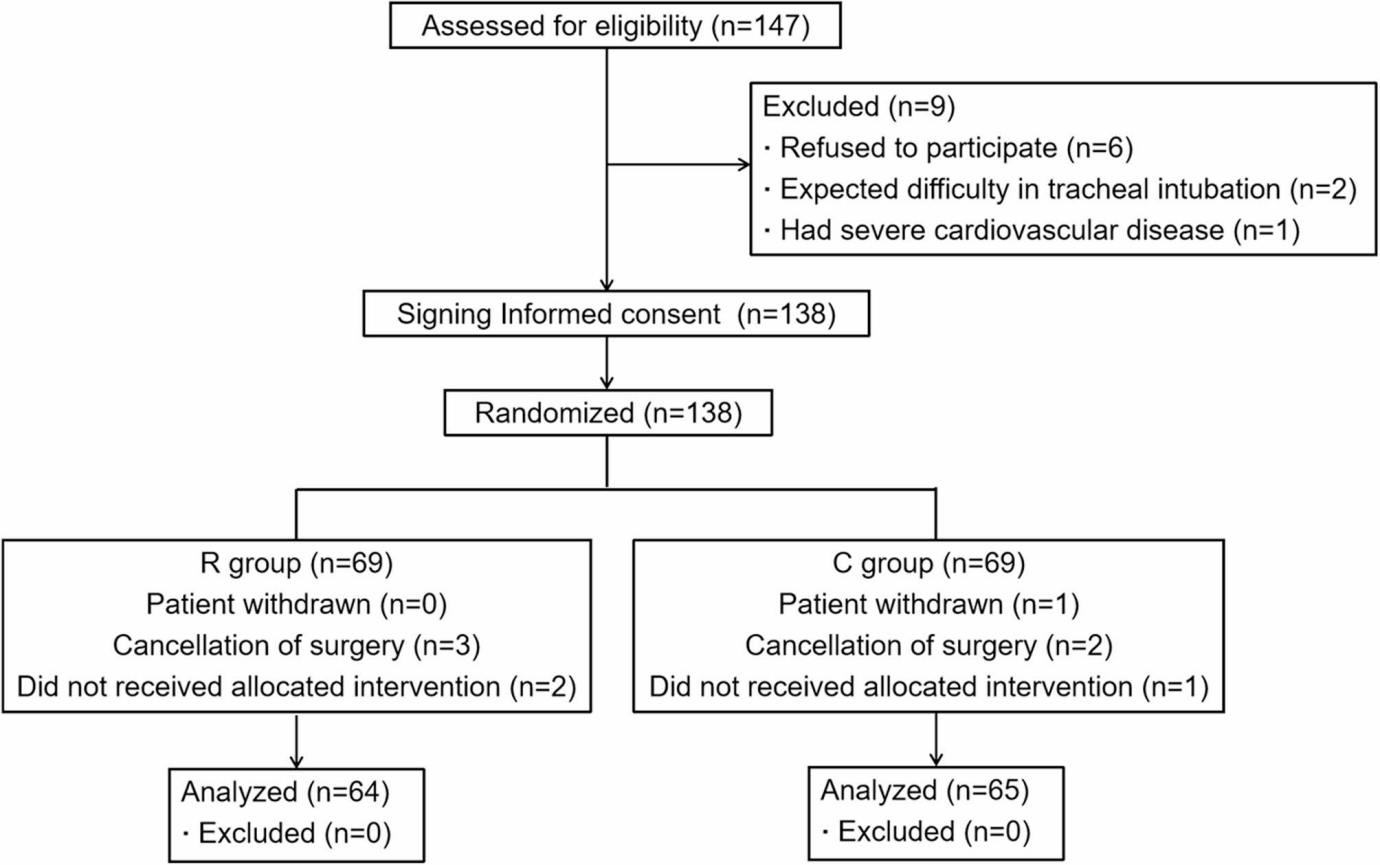
CONSORT flow chart of the study

We screened 147 children for eligibility (Fig. 1). Exclusion criteria were applied to 9 patients. The final cohort (*n* = 138) was randomized 1:1. Finally, 129 pediatric patients aged 1–12 years completed the study. Among them, 64 and 65 patients were included in groups R and C, respectively. Table 1 shows the demographic data and surgical characteristics. There were no significant differences in patient characteristics. The operative times of the patients were 62.4 ± 31.9 min in group R and 58.8 ± 31.9 min in group C (*p* = 0.494). The types of surgery were not significantly different between the two groups.

**Table 1:** Patient characteristics

Variables	Group *R* (*n* = 64)	Group C (*n* = 65)	*p* value
Age (years)	4.4 ± 2.9	4.6 ± 2.6	0.741
Sex			0.504
Male	47 (73.4)	51 (78.5)	
Female	17 (26.6)	14 (21.5)	
Weight (kg)	20.3 ± 9.0	20.8 ± 9.2	0.558
BMI (kg·m^−2^)	15.7 ± 1.6	15.8 ± 1.5	0.677
ASA			
I	60 (93.8)	63 (97.0)	0.623
II	4 (6.3)	2 (3.1)	0.623
Mallampati score			
I	28(43.8)	29(44.6)	0.921
II	34(53.1)	33(50.8)	0.789
III	2(3.1)	3(4.6)	0.661
IV	0(0.0)	0(0.0)	-
Laryngoscopy			
Direct laryngoscopy	50 (78.1)	54 (83.1)	0.477
Videolaryngoscopy	14 (21.9)	11 (16.9)	0.477
Duration of surgery (min)	62.4 ± 31.9	58.8 ± 31.9	0.494
Type of surgery			
Urological	35 (54.7)	34 (52.3)	0.786
Ear, nose, and throat	15 (23.4)	17 (26.2)	0.721
Orthopaedic	8 (12.5)	10 (15.4)	0.636
Digestive	4 (6.3)	3 (4.6)	0.983
Stomatological	2 (3.1)	1 (1.5)	0.989

*BMI* body mass index, *ASA* American Society of Anaesthesiologists

Data are presented as means ± SDs, n (%)

### Primary outcome

In the randomized population, the incidence of successful tracheal intubation on the first attempt with no adverse events in Group R (87.5%, *n* = 56) was greater than that in Group C (60.0%, *n* = 39) (OR, 4.7; 95% CI = 1.9–11.4; *p* < 0.001) (Table 2). In terms of the primary outcome, the incidence of successful intubation on the first attempt, regardless of adverse events, was not significantly different between the two groups (*p* = 0.283). The incidence of body movement during intubation in group C was greater than that in group R (*p* = 0.001). The incidences of choking during intubation and arrhythmia requiring intervention (bradycardia) during intubation were not significantly different between the two groups (*p* = 0.121, and *p* = 0.989, respectively).

**Table 2:** Outcomes

Variables	Group *R* (*n* = 64)	Group C (*n* = 65)	*p* value
Primary outcome			
Successful intubation on the first attempt with no adverse events	56 (87.5)	39 (60.0)	< 0.001
Details of the primary outcome			
Successful intubation on the first attempt	62 (96.9)	59 (90.8)	0.283
Choking during intubation	3 (4.7)	8 (12.3)	0.121
Body movement during intubation	6 (9.4)	19 (29.2)	0.001
Arrhythmia requiring intervention during intubation	2 (3.1)	1 (1.5)	0.989
Secondary outcomes			
Time from induction to successful intubation^a^ (s)	147.2 ± 30.5	202.2 ± 46.4	< 0.001
Desaturation occuring during induction to successful intubation^b^	0 (0.0)	1 (1.5)	1.000
Laryngospasm or bronchospasm	0 (0.0)	2 (3.0)	0.496

Data are presented as means ± SDs, n (%)

a Time from induction to successful intubation is defined as the time from the start of drug administration to successful placement of the endotracheal tube in the glottis

b Desaturation is defined as a SpO2 < 95%

### Secondary outcomes

Considering the indicators related to tracheal intubation, the time from induction to successful intubation was shorter in group R than in group C (*p* < 0.001) (Table 2). The incidence of desaturation did not significantly differ between the groups (*p* = 1.000). The incidence of laryngospasm or bronchospasm did not significantly differ between the groups (*p* = 0.496).

**Fig. 2:**
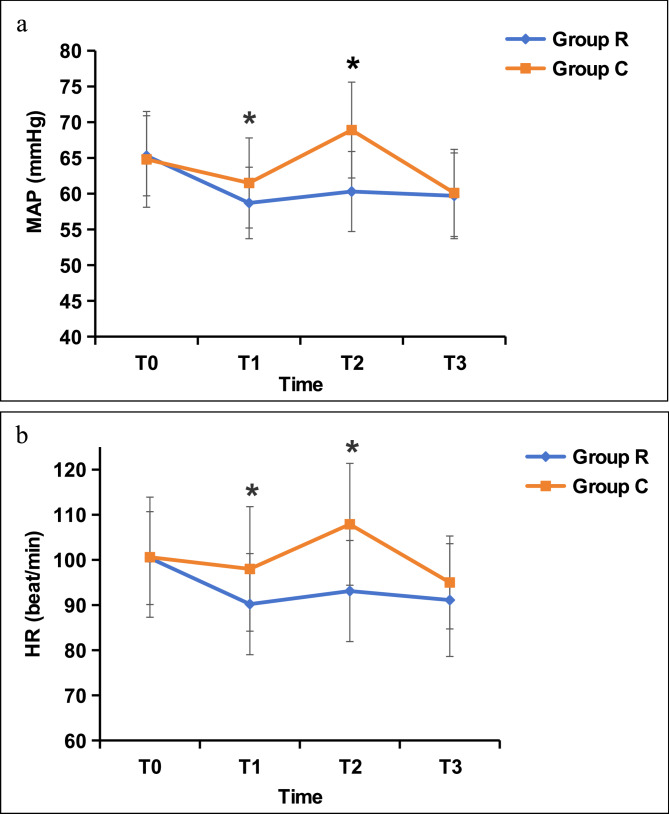
Mean arterial pressure and heart rate at each time point in the two groups. T0: baseline; T1: immediately before intubation; T2: immediately after intubation; T3: 5 min after intubation

The hemodynamic values at each time point in the two groups are presented in Fig. 2. Both the MAP and HR were significantly different between the two groups according to repeated-measures ANOVA (group effects, *p* = 0.001 and *p* < 0.001). The MAP and HR changed significantly with time (*p* < 0.001), and a significant interaction with time × group (*p* < 0.001) was detected in groups R and C. Figure 2a shows that there were significant differences in the MAP between the two groups at T1 and T2 (*p* < 0.01), with the MAP in group R being significantly lower than that in group C. Figure 2b shows that there were significant differences in the HR between the two groups at T1 and T2 (*p* < 0.01), with the HR in group R being significantly lower than that in group C.

## Discussion

Compared with conventional general anesthesia induction drugs, the combined use of low-dose remifentanil in children is effective at improving the success rate of first tracheal intubation without adverse events (87.5% vs. 60.0%, *p* < 0.001).

The type and dosage of sedatives and muscle relaxants are the key factors to be considered for successful tracheal intubation. As liver and kidney metabolism in children is inferior to that in adults, cisatracurium is used relatively more frequently because of its short onset of action and Hofmann elimination and its low dependence on liver and kidney function [13, 14]. Propofol is also the most commonly used drug for intravenous anesthesia induction in children because of its rapid onset, rapid elimination and low likelihood of accumulation[15, 16]. However, as previously noted, anesthetics employed in rapid sequence intubation (RSI) vary significantly in their onset times to achieve adequate neuromuscular blockade depth for safe airway management. The accelerated induction protocol carries inherent risks of physiological responses, including patient movement and activation of airway reflexes such as coughing or laryngospasm. While these adverse events can be mitigated through transitional assisted ventilation, this requirement introduces a trade-off that undermines the primary advantage of RSI—the immediate establishment of definitive airway control. The consequent delay in securing the airway creates a paradoxical clinical scenario where the intervention intended to expedite protection instead prolongs the at-risk period for pulmonary aspiration. Therefore, this study aimed to explore combinations of other drugs to avoid the occurrence of related adverse events. Previous studies have shown that the use of remifentanil during the induction of anesthesia facilitates the rapid achievement of optimal intubation conditions [17, 18] The most commonly recommended dose for acceptable intubating conditions without a neuromuscular blocker is remifentanil (3–4 µg/kg) [17–19] However, increases in the dosage of remifentanil increase the risks of hypotension and bradycardia [8–20]. The dose of remifentanil can be reduced when neuromuscular blocking agents are used. Under these conditions, the recommended dose of remifentanil is mostly 0.5–2 µg/kg [21, 22]. Considering its safety and efficacy, we chose a dose of 1 µg/kg. As expected, we found that remifentanil (1 µg/kg) combined with other anesthetic drugs can reduce the occurrence of related adverse events during intubation (group R: group C = 87.5%:60.0%, *p* < 0.001), which ensures better conditions for intubation.

If the sedation depth of anesthesia is inadequate during tracheal intubation, it may cause choking and body movement, and in severe cases, it may lead to adverse events such as laryngospasm, which may affect patient safety. Previous data have suggested that respiratory events are more common in children than in adults [23], thus leading to a range of related problems, such as an increase in the number of claims and an increased financial burden. This is also related to suboptimal conditions for tracheal intubation. In this study, although the use of low-dose remifentanil (1 µg/kg) did not affect the success rate of tracheal intubation (group R: group C = 96.9%:90.8%, *p* = 0.283), it effectively reduced adverse events related to tracheal intubation (group R: group C = 87.5%:60.0%, *p* < 0.001). Although most of the adverse events were mild and did not affect normal intubation, and were able to meet the needs of daily work, the most common adverse events were body movement, which also indicated that the depth of anesthesia or degree of muscle relaxation in group C was insufficient. For inexperienced anesthesiologists or patients with special circumstances requiring early airway protection, such adverse events may have serious consequences. For example, in children with asthma, even mild airway irritation or choking may cause bronchospasm. In contrast to the adverse effects of high-dose remifentanil [17–24], such as bradycardia or blood pressure reduction, there was no increased risk of arrhythmia in our study (group R: group C = 3.1%:1.5%, *p* = 0.989). Therefore, the use of remifentanil during anesthesia induction is able to improve intubation conditions while ensuring the safety of clinical work, which is also consistent with the conclusions of previous studies [21].

The results of this study revealed that the time from the beginning of anesthesia induction to successful tracheal intubation in group R was shorter than that in group C, indicating that the combination of low-dose remifentanil can shorten the time of tracheal intubation. In some special cases, such as in children with gastric satiety requiring rapid sequential induction, an alternative induction scheme may be necessary if the child has contraindications to the use of succinylcholine or rocuronium. In our study, two patients in group C developed laryngospasm or bronchospasm, but none did in group R. Although the results of the two groups were not statistically significant (*p* = 0.496), this may also be due to the small sample size, which was also confirmed in some studies [25, 26]. In addition, remifentanil is rapidly metabolized and does not affect postoperative recovery.

In addition, this study evaluated the hemodynamics of patients during intubation. Tracheal intubation can cause a sympathetic adrenergic response and hemodynamic disorders, leading to adverse events such as hypertension and arrhythmia and even more serious cardiovascular and cerebrovascular events [27, 28]. Mild hemodynamic changes are tolerable in healthy children but can be fatal in children with congenital heart disease, impaired cardiac function, or vascular malformations. In this study, the changes in blood pressure and HR before and after intubation in group R were smaller than those in group C, which is consistent with previous study [22]. These results indicate that 1 µg/kg remifentanil can effectively reduce the adrenergic response, which is also consistent with the results of previous studies [7, 8].

### Limitations

This study has several limitations. First, the study population included ASA I-II children aged 1–12 years. Further studies are needed to confirm the safety and efficacy of the remifentanil dose used in patients of other ages or with other comorbidities. Second, this was a single-center study, and the rate of successful intubation was higher than that in some areas [29, 30] [12]. However, for centers with little experience in pediatric anesthesia, mild choking or body movement can lead to tracheal intubation failure, so the actual rate of successful tracheal intubation may be lower. Third, long-term follow-up to assess the impact of remifentanil on postsurgical recovery is lacking.

## Conclusions

In pediatric patients undergoing elective tracheal intubation, the use of low-dose remifentanil in combination with cisatracurium significantly increased the probability of successful first-attempt intubation without adverse events compared with the cisatracurium alone. In addition, it can shorten the time to achieve satisfactory tracheal intubation conditions and reduce the cardiovascular response to tracheal intubation.

## Data Availability

The datasets used and/or analyzed during the current study are available from the corresponding author on reasonable request.

## Abbreviations

ASA: American society of anesthesiologists
BIS: Bispectral index
ECG: Electrocardiography
NIBP: Noninvasive blood pressure
SpO_2_: Pulse oxygen saturation
MAP: Mean arterial pressure
HR: Heart rate
BMI: Body mass index
RSI: Rapid sequence intubation
